# Editorial: Trends on valorization of agri-food waste through green technologies

**DOI:** 10.3389/fnut.2025.1615743

**Published:** 2025-05-07

**Authors:** Jelena Vladić, Nataša Nastić

**Affiliations:** ^1^LAQV, REQUIMTE, Faculdade de Ciências e Tecnologia, Universidade Nova de Lisboa, Caparica, Portugal; ^2^Faculty of Technology, University of Novi Sad, Novi Sad, Serbia

**Keywords:** green solvents, food waste and by-products, biorefinery, valorization by-product, food processing

The generation of agri-food waste and by-products across multiple stages, including harvest, post-harvest processing, industrial production, distribution, and storage, presents a significant environmental, economic, and social challenge. This process affects the sustainability of food production systems worldwide (1). In response to the urgent need for solutions, this Research Topic focuses on applying various green strategies to transform agri-food residues into valuable resources and to improve the efficiency of resource use.

The growing importance of green chemistry principles has driven innovations that reduce water and energy consumption, limit the use of hazardous materials, and promote environmental sustainability. Since the late 20^th^ century, research and technological development have increasingly focused on creating environmentally compliant processes to meet industrial and societal needs (2).

This Research Topic gathers original research that advances green extraction methods, bioconversion strategies, and new applications for by-products. These studies emphasize the role of different green strategies in reshaping food production chains and moving toward a circular economy model.

Xu et al. investigated the use of agro-forest waste as an alternative substrate for the cultivation of *Lentinus edodes* (shiitake mushroom). By incorporating pruning residues from Korshinsk peashrub, seabuckthorn, and goji berry plants, the study assessed the effects on mycelial growth, mushroom yield, and nutritional quality. Specific waste-based formulations supported effective mushroom production and improved the nutritional profile, especially in terms of protein, fiber, amino acids, and organic acids. Principal Component Analysis identified optimal substrate combinations that ensured high yields and enhanced quality. The study shows that replacing traditional oak sawdust with agro-forest residues can reduce production costs and contribute to environmental sustainability in mushroom farming.

In another contribution, Dina et al. developed a valorization pathway for oregano and thyme by-products generated during the olive oil aromatization process. Using microwave-assisted extraction, the authors demonstrated that post-processing herbal residues still contain significant amounts of phenolic compounds and antioxidant compounds such as carvacrol and thymol. Chemical profiling and antioxidant evaluations confirmed the potential of these by-products as sources of high-value ingredients for nutraceuticals, cosmeceuticals, and animal feed. This work supports the broader effort to create sustainable uses for agro-industrial waste within Mediterranean food systems.

Scheibenzuber et al. addressed consumer acceptance of food products enriched with ingredients derived from food industry by-products. Focus groups conducted in Germany, Italy, Romania, and Norway revealed a generally positive attitude toward using by-products, particularly due to perceived environmental and health benefits. Preferred product categories included bakery, dairy, and meat products. However, concerns regarding food safety, allergens, and chemical contaminants emerged as barriers to acceptance. The study highlights the importance of clear communication and strong food safety assurances to promote consumer trust and encourage the adoption of innovative products made from by-products.

Shi et al. focused on improving the bioactivity of angiotensin I-converting enzyme (ACE) inhibitory peptides obtained from tiger nut (*Cyperus esculentus*) protein hydrolysates. Through a plastein reaction catalyzed by trypsin, the researchers produced a modified peptide product with enhanced ACE inhibitory activity compared to the original hydrolysate. Structural changes and aggregation phenomena were confirmed through Scanning Electron Microscopy, Fourier-transform infrared spectroscopy, X-ray powder diffraction, and fluorescence spectroscopy analyses. The results suggest that the plastein reaction is an efficient method to enhance the bioactivity of plant-derived peptides and support the development of natural ACE inhibitors for potential use in functional foods and nutraceuticals.

The contributions featured in this Research Topic demonstrate the diverse opportunities for applying green strategies to valorize agri-food waste and by-products. Advances in extraction techniques, biotechnological processing, consumer insights, and bioactivity enhancement point toward a future in which food waste represents a valuable resource. Interdisciplinary collaboration will be essential to fully exploit these opportunities and to build more efficient and sustainable food production systems.
